# Postnatal Bonding in the First Year After Birth: The Role of Maternal Depression, Resilience, and Anxiety During the COVID-19 Pandemic and War-Related Stress—A Prospective Cohort Study

**DOI:** 10.3390/jcm15041451

**Published:** 2026-02-12

**Authors:** Ewelina Barszcz, Maksymilian Kamil Plewka, Aleksandra Margulska, Dominika Kędzia, Klaudia Sójta, Katarzyna Nowakowska-Domagała, Dominik Strzelecki, Oliwia Gawlik-Kotelnicka

**Affiliations:** 1 Department of Affective and Psychotic Disorders, Medical University of Lodz, Czechosłowacka 8/10, 92-216 Łódź, Polanddominik.strzelecki@umed.lodz.pl (D.S.); 2 Department of Electrocardiology, Central Teaching Hospital, Medical University of Lodz, Pomorska 251, 92-213 Łódź, Poland; 3 Department of Child and Adolescent Psychiatry, Medical University of Lodz, Czechosłowacka 8/10, 92-216 Łódź, Poland; aleksandra.margulska@umed.lodz.pl; 4 Department of Clinical Psychology and Psychopathology, Institute of Psychology, University of Lodz, al. Rodziny Scheiblerów 2, 90-128 Łódź, Poland; katarzyna.nowakowskadomagala@now.uni.lodz.pl

**Keywords:** postnatal bonding, Postpartum Bonding Questionnaire, COVID-19, war, perinatal depression, Edinburgh Postnatal Depression Scale, resilience

## Abstract

**Background:** Postnatal bonding reflects the early emotional relationship between a mother and her infant and is shaped by psychological and perinatal factors. This study examined associations between postnatal bonding and maternal depressive symptoms, resilience, labor anxiety, and sociodemographic and health-related variables, as well as anxiety related to the COVID-19 pandemic, the war in Ukraine, and global events. **Methods:** This prospective cohort study included and followed 150 pregnant women in Poland from pregnancy to 12 months postpartum. Assessments were conducted during pregnancy and at 4–6 weeks, 6 months, and 12 months after delivery. Postnatal bonding was assessed using the PBQ, depressive symptoms with the EPDS, labor anxiety with the LAQ, resilience with the KOP-26, and anxiety related to external stressors with study-specific questionnaires. Non-parametric analyses were performed with a significance level of *p* < 0.05. **Results:** Postnatal bonding difficulties were most prevalent at 4–6 weeks postpartum (26.1%) and decreased over time. Maternal depressive symptoms showed the strongest and most consistent associations with bonding difficulties, whereas higher resilience, particularly in family relations and social competence, was associated with better bonding outcomes. Labor anxiety was weakly associated with bonding only in the early postpartum period, while no associations were found with sociodemographic characteristics or anxiety related to external societal stressors. **Conclusions:** Maternal depressive symptoms and resilience emerged as the key factors associated with postnatal bonding quality, highlighting their importance as targets for early identification and intervention.

## 1. Introduction

Pregnancy marks a transformative period in a woman’s life, characterized by significant hormonal, emotional, and social role changes [1]. Postnatal bonding (PB) describes the emotional connection between a mother and her infant, characterized by joy and fulfillment from their interactions. This bond fosters the infant’s emotional communication while enhancing the mother’s confidence, competence, and adaptability in meeting her child’s needs and embracing the maternal role [2]. An adequate mother–child bond constitutes a foundation for the child’s socio-emotional development and significantly influences maternal sensitivity, the effectiveness of parenting practices, and overall parental competence. In contrast, reduced maternal sensitivity and weaker bonding are associated with insecure attachment and an increased risk of depression, anxiety, and socio-emotional developmental disturbances [3,4].

The formation of PB is influenced by various demographic, perinatal, and psychological factors [5]. Among the demographic factors examined in research as potentially related to the formation of PB are maternal age, educational level, and birth order of the child [6,7,8,9]. Medical and obstetric factors are also significantly associated with the formation of maternal–infant bonding. Although pregnancy and delivery complications have been linked to disturbances in bonding, the available evidence remains inconclusive [5,7,8,10]. Breastfeeding and skin-to-skin contact are considered important factors supporting the establishment and strengthening of the maternal–infant bond, whereas the absence of breastfeeding or its early discontinuation may constitute a risk factor for persistent bonding difficulties [10,11]. Psychological aspects also play a crucial role. One of the key psychological factors associated with the formation of the mother–child bond is the presence and quality of partner support [12]. Partner support reduces stress levels in both the mother and the child after birth, whereas greater disappointment with the partner has been linked to weaker bonding with the child [13,14]. Support from the father also plays a significant role in the child’s psychosocial development at later stages of life [15]. These diverse factors highlight the multifaceted nature of the bonding process between mother and child.

Perinatal depression (PD), defined in the Diagnostic and Statistical Manual of Mental Disorders (DSM-5) as a depressive episode occurring during pregnancy or within four weeks postpartum, may weaken PB and hinder the establishment of an emotional connection with the child [16,17]. The mechanisms linking PD to bonding disturbances include reduced maternal sensitivity to fetal activity and early caregiving difficulties, such as problems with breastfeeding or excessive infant crying. These challenges may intensify depressive symptoms, creating a vicious cycle that further undermines maternal confidence and sensitivity toward the child [18,19]. Women experiencing postpartum depressive symptoms report lower satisfaction with motherhood, elevated stress levels, and pronounced discrepancies between prenatal expectations and postnatal reality [14].

Labor anxiety constitutes a distinct form of anxiety occurring during the perinatal period [20]. Factors contributing to negative subjective birth experiences, such as unforeseen medical complications, intense pain and loss of control, feelings of guilt, and the quality of intrapartum care, may indirectly increase maternal stress and anxiety and contribute to additional perinatal complications that are negatively associated with PB [21]. Moreover, elevated maternal anxiety during labor may disrupt early mother–infant interactions by reducing maternal sensitivity, thereby hindering the development of secure attachment and effective bonding processes [22].

Resilience is the ability to adapt positively and to maintain or regain mental health despite adversity, shaped by the interplay of personal, biological, and environmental factors [23]. During the perinatal period, resilience serves as a protective function and acts as a key intrapersonal regulatory mechanism supporting the stabilization of maternal emotions in challenging situations [24,25]. It also mediates the relationship between maternal attachment styles and mother–infant bonding, thereby facilitating the formation of secure emotional connections. Mothers with higher levels of resilience demonstrate a greater capacity to establish strong and enduring bonds with their infants [26].

In addition to individual stressors examined in previous studies, the potential role of macro-social, external stressors in the psychological functioning of women during the perinatal period should also be considered, as these affect entire societies. In Poland, recent years have seen significant stressors, including the COVID-19 pandemic and the outbreak of war in neighboring Ukraine. The COVID-19 pandemic significantly disrupted pregnancy and the postpartum period through restrictions in obstetric care, reduced access to support, increased social isolation, and heightened anxiety related to the risk of infection, which together contributed to poorer maternal mental health and a higher prevalence of perinatal depression [27,28,29]. Although no significant differences in PB disturbances were observed before and during the pandemic, elevated levels of depression and anxiety suggest a possible indirect adverse effect of the pandemic on the development of the mother–child relationship [30,31,32]. Research on mother–child relationships has been conducted predominantly under peaceful conditions. Although Poland is not directly affected by armed conflict, as a country neighboring the war in Ukraine, it faces significant economic, social, and psychological consequences [33]. Ongoing exposure to war-related information, involvement in humanitarian aid for refugees, and rising inflation contribute to heightened uncertainty, anxiety, and psychological burden. Recent studies indicate increased levels of anxiety and depression in the general population; however, research focusing on the mental health of perinatal women in this context remains limited, particularly regarding the impact of war-related stress on PB [34].

This study aimed to examine the relationships between PB and selected factors, including resilience, perinatal depression, labor anxiety and sociodemographic and health-related variables. Additionally, the study sought to explore associations between anxiety related to the COVID-19 pandemic and the economic situation in Poland and the quality of PB. It was hypothesized that higher levels of stress related to the COVID-19 pandemic, war, and economic crisis, as well as lower resilience, higher levels of depressiveness, and greater childbirth anxiety would be associated with lower PB quality.

## 2. Material and Methods

### 2.1. Design and Population

This prospective cohort study was conducted in two phases among the perinatal population in Poland. The first phase was carried out between 17 February 2021 and 25 November 2021 during the COVID-19 pandemic, while the second phase took place from 2 June 2022 to 11 April 2023, amidst economic instability and geopolitical tensions resulting from the armed conflict in Ukraine. Participation in the study was voluntary, and inclusion criteria required participants to be pregnant when completing the survey. Out of all the pregnant Polish women who took part in the first and second stages of the study, the present analyses included only participants who completed at least one later postpartum assessment (6 and/or 12 months postpartum).

All participants were informed about the study’s objectives and the principles of confidentiality regarding the use of the collected data. Informed electronic consent was obtained before participants completed the online questionnaires. The surveys were administered online using Google Forms. Postpartum assessments were distributed within several days of the target time points corresponding to approximately 4–6 weeks, 6 months, and 12 months after delivery. Responses across time points were linked using participants’ email addresses provided at enrollment and subsequently pseudonymized to ensure confidentiality. They were also informed of their right to withdraw from the study at any time. Recruitment was conducted through social media, parenting portals, and leaflets distributed in obstetrics and gynecology clinics.

The study received ethical approval from the Bioethics Committee of the Medical University of Lodz (No. RNN/39/21/KE, 9 February 2021). To ensure participant privacy, all personal data were pseudonymized. The manuscript has been prepared according to the STROBE State [35].

Due to participant attrition and incomplete follow-up across study phases, the number of participants contributing PBQ data varied between assessment points. Initially, 150 women were enrolled in the study, of whom PBQ data were available for 92 participants at T3, 110 participants at T4, and 115 participants at T5. Detailed information on participant flow and attrition across assessment points is presented in Supplementary Table S1. Baseline comparisons between completers and non-completers are shown in Supplementary Table S2. Significant differences were observed in previous psychiatric treatment history (χ^2^ = 7.480, *p* = 0.006), with a higher proportion of prior treatment among completers. In addition, PBQ scores at T4 differed significantly between groups (U = 825.0, Z = 2.325, *p* = 0.020), whereas no other sociodemographic, clinical, or psychological variables showed significant differences.

### 2.2. Methods

Several standardized and study-specific instruments were used to assess psychological variables relevant to the study objectives.

The Postpartum Bonding Questionnaire (PBQ) developed by I. F. Brockington served as the primary measure of the parent–infant relationship, with a specific focus on identifying potential bonding difficulties during the postpartum period. The instrument was originally designed for use alongside the Edinburgh Postnatal Depression Scale as a screening tool to facilitate the early identification of mother–infant bonding disorders. This 25-item tool comprises four subscales: mother–infant relationship disorders, rejection and pathological anger, infant-focused anxiety, and incipient abuse. Each item is rated on a 6-point Likert scale ranging from “always” to “never”, yielding a total score from 0 to 125, with higher scores indicating greater bonding difficulties. The cumulative total score is used to screen for general bonding disorders (cut-off ≥ 26) and severe bonding disturbances (cut-off ≥40), and cut-off scores have also been proposed for each subscale [36,37].

Other psychological constructs were assessed using additional measures. The Edinburgh Postnatal Depression Scale (EPDS) evaluated depressive symptoms during pregnancy and postpartum. The Labor Anxiety Questionnaire (LAQ) measured childbirth-related anxiety, and the Resilience Measure Questionnaire (KOP-26) assessed resilience across family, personal, and social domains. Furthermore, original tools were developed for this study: the COVID-19 Pandemic-Related Anxiety Questionnaire (CRAQ), administered in the first phase, and the Global Situation Anxiety Questionnaire (GSAQ) and War Anxiety Questionnaire (WAQ), both used in the second phase to examine anxiety in response to societal and geopolitical stressors. The development of these instruments was informed by a review of the relevant literature and clinical experience, with items designed to capture context-specific anxiety symptoms. The full item content of the CRAQ, GSAQ, and WAQ, along with scoring procedures, is provided in the Supplementary Material. The internal consistency of the scales was assessed using Cronbach’s alpha coefficients, which indicated acceptable reliability (see Supplementary Material). However, formal psychometric validation procedures, such as factor analysis or external validation against established measures, have not yet been conducted. Therefore, findings based on these instruments should be interpreted as exploratory.

Data were collected at five time points: T1 (<33 weeks of gestation), T2 (33–37 weeks of gestation), T3 (4 weeks postpartum), T4 (6 months postpartum), and T5 (12 months postpartum). Information on the questionnaires and scales administered at each time point is provided in Table 1.

### 2.3. Data Analysis

Statistical analyses were performed using STATISTICA 13.1 (TIBCO Software Inc., Palo Alto, CA, USA). For continuous variables, descriptive statistics were calculated, including mean, standard deviation, and minimum and maximum values. Discrete variables were described using counts and percentages. The Shapiro–Wilk test was applied to assess the normality of distribution. The Mann–Whitney U test and the Kruskal–Wallis test were used for intergroup comparisons. Differences in psychometric scores between two time points were assessed with the Wilcoxon signed-rank test, and for multiple time points with the Friedman test. Associations between psychometric scores and sociodemographic or clinical variables were analyzed using Spearman’s rank correlation coefficients.

In addition, multiple linear regression models were constructed to examine independent predictors of PBQ scores at postpartum time points. Maternal depressive symptoms (EPDS), resilience (KOP-26), and labor anxiety (LAQ) were included as primary predictors, along with maternal age, parity, education level, and history of psychiatric treatment as covariates. Regression assumptions were evaluated using residual diagnostics to confirm linearity, homoscedasticity, and normality. Analyses were conducted using complete-case data, and no imputation of missing values was performed.

Furthermore, to analyze longitudinal changes in maternal–infant bonding and identify significant predictors across repeated postpartum measurements, a Generalized Estimating Equation (GEE) model with a Poisson distribution and an exchangeable correlation structure was employed. The GEE analysis was performed using Python (version 3.10) with the statsmodels library.

A significance level of *p* < 0.05 was adopted.

## 3. Results

### 3.1. Sample Characteristics

The study group comprised 150 pregnant Polish women who met the inclusion criteria. Most participants reported higher education (91%), defined as completed university-level education, and 63% resided in urban areas with populations over 100,000. At the time of the study, 51% of respondents were nulliparous. Pregnancy complications were reported by 23% of participants, and 32% indicated a history of psychiatric treatment. Detailed demographic data are presented in the Supplementary Table S7.

EPDS scores showed a peak in depressive symptoms (EPDS ≥ 14) at 4–6 weeks postpartum in 30% of participants, decreasing to 11% after one year. Childbirth-related anxiety was reported by 51% of women before 33 weeks’ gestation. Additionally, 23% reported significant anxiety related to the ongoing war, and 22% expressed considerable anxiety about global geopolitical issues. At least moderate anxiety related to the COVID-19 pandemic was observed in 56% of participants. Detailed depression and anxiety data are shown in the Supplementary Table S8.

An analysis of resilience revealed that 53% of participants demonstrated low overall resilience. Detailed results, including resilience subscales scores, are presented in the Supplementary Table S9.

### 3.2. Postnatal Bonding

**Figure 1:**
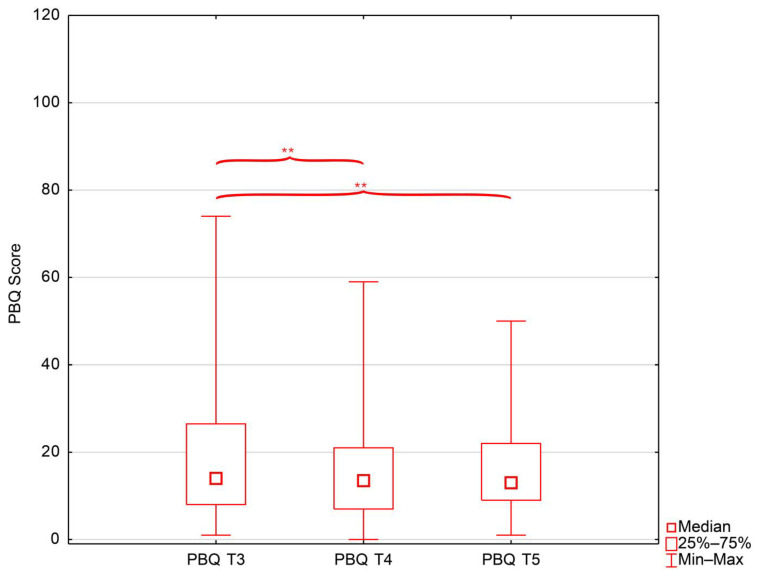
Boxplot illustrating changes in postpartum bonding across time points postpartum. Abbreviations: PBQ—Postpartum Bonding Questionnaire; T3—4–6 weeks postpartum; T4—6 months postpartum; T5—12 months postpartum; **—statistical significance (*p* < 0.05).

Postnatal bonding difficulties were most pronounced at 4–6 weeks postpartum, with 26.1% of mothers scoring in the impaired (PBQ ≥ 26) or severely impaired (PBQ ≥ 40) range; this proportion declined to 14.5% at 6 months and 15.6% at 12 months. A comparison of PBQ scores across time points showed significant differences (Friedman test: (χ^2^ (2) = 14.55, *p* < 0.001; W = 0.12), with mean ranks decreasing from 2.37 at 4–6 weeks postpartum to 1.91 at 6 months and 1.72 at 12 months (Figure 1). Pairwise analyses confirmed significant reductions in PBQ scores between 4 and 6 weeks and 6 months (n = 79, T = 964.5; Z = 3.01; *p* = 0.003) and between 4 and 6 weeks and 12 months (n = 66, T = 691.0; Z = 2.65; *p* = 0.008), while the difference between 6 and 12 months was not significant (n = 72, T = 1213.0; Z = 0.57; *p* = 0.571).

Difficulties were mainly reflected in the mother–infant relationship disorders subscale, whereas scores for rejection/pathological anger and infant-focused anxiety remained consistently low; indicators of incipient abuse were virtually absent at all time points. Detailed results by time point and subscale are presented in Table 2.

PBQ scores showed no associations with anxiety related to war, global events, or the COVID-19 pandemic at any measurement point. At 4–6 weeks postpartum, PBQ showed a weak positive correlation with labor anxiety reported before 33 weeks of gestation and a moderate positive correlation with labor anxiety reported between 33 and 37 weeks; no significant associations were observed at later postpartum assessments. Resilience was consistently negatively associated with PBQ. The overall KOP-26 score demonstrated weak-to-moderate inverse correlations with PBQ at all postpartum assessments. Among the subscales, family relations showed the strongest and most stable associations. Depressive symptoms exhibited strong and consistent associations with PBQ. EPDS scores collected during pregnancy (T1–T2) demonstrated weak-to-moderate positive correlations with PBQ at 4–6 weeks postpartum, with only the T2 assessment remaining weakly associated at 6 months. Postpartum EPDS scores (T3–T5) were moderately and positively correlated with PBQ at all corresponding time points. No consistent associations were observed between EPDS scores and parity or mode of delivery across the assessed time points. Detailed correlation coefficients are presented in Table 3.

**Figure 2:**
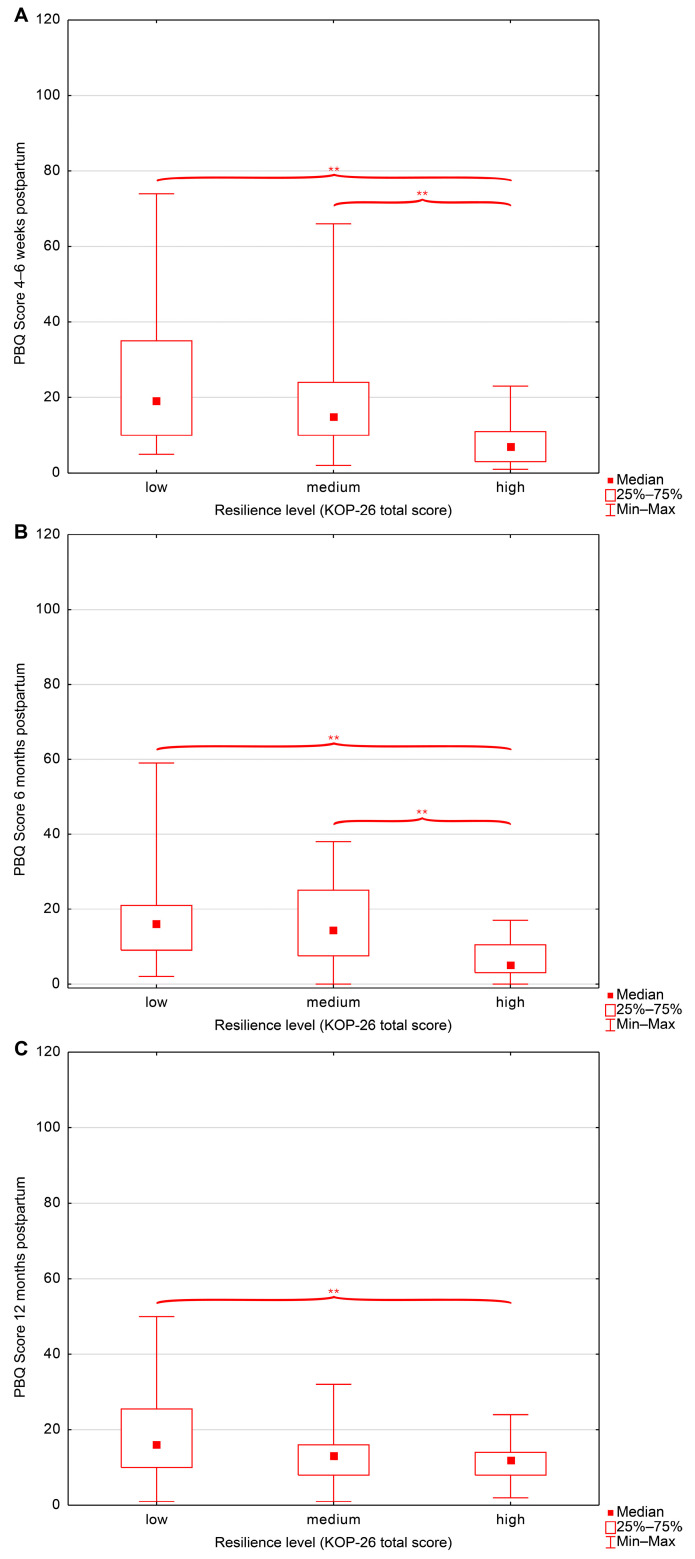
Postpartum Bonding Questionnaire (PBQ) scores across resilience levels (KOP-26) at (**A**) 4–6 weeks postpartum, (**B**) 6 months postpartum, and (**C**) 12 months postpartum. Abbreviations: PBQ—Postpartum Bonding Questionnaire, KOP26—Resilience Measure Questionnaire; **—statistical significance (*p* < 0.05).

In contrast, group comparisons across overall resilience levels (low, medium, high) and resilience subscales revealed significant differences in PBQ scores at all postpartum time points (Table 4, Figure 2).

Across all postpartum assessments, women with high overall resilience consistently reported fewer postnatal bonding difficulties than those with low or moderate resilience. At 4 weeks postpartum, PBQ scores were the lowest in the high-resilience group compared with both the low- and moderate-resilience groups. A similar pattern was observed at 6 months postpartum, with lower PBQ scores in the high-resilience group relative to the low- and moderate-resilience groups. At 12 months postpartum, PBQ scores remained significantly lower among women with high resilience compared with those with low resilience. Comparable patterns were observed for the family relations subscale. Women with high family-related resilience exhibited lower PBQ scores at 4 weeks postpartum than those with low or moderate resilience, and this effect persisted at 6 months postpartum. At 12 months postpartum, PBQ scores in the high-resilience group remained lower than those in the moderate-resilience group. In contrast, the personal competence subscale differentiated PBQ scores only at 6 months postpartum, with lower scores observed in the high-resilience group compared with both the low- and moderate-resilience groups. Social competence showed a broader pattern: at 6 months postpartum, PBQ scores were lower in the high-resilience group than in the low-resilience group, whereas at 12 months postpartum, PBQ scores were significantly lower in both the high- and moderate-resilience groups compared with the low-resilience group.

**Figure 3:**
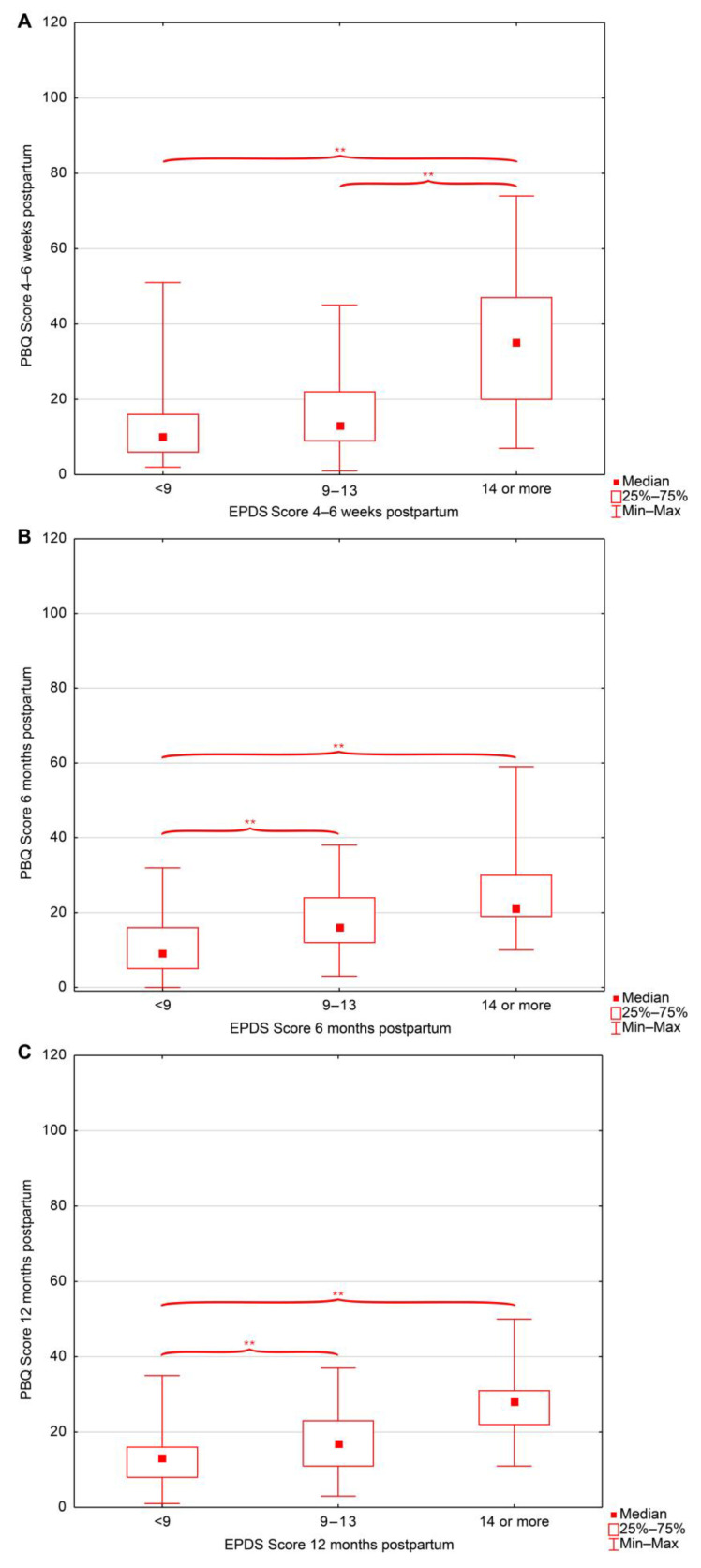
Postpartum Bonding Questionnaire (PBQ) scores across depressive symptom levels (EPDS) at (**A**) 4–6 weeks postpartum, (**B**) 6 months postpartum, and (**C**) 12 months postpartum. Abbreviations: PBQ—Postpartum Bonding Questionnaire, EPDS—the Edinburgh Postnatal Depression Scale; **—statistical significance (*p* < 0.05).

With respect to the severity of depressive symptoms assessed using the EPDS, significant differences in PBQ scores were observed depending on the timing of EPDS assessment (Table 5, Figure 3).

Group comparisons based on depressive symptom severity, classified according to EPDS scores (low: <9, moderate: 9–13, high: ≥14), revealed consistent differences in postnatal bonding across postpartum assessments. When depressive symptoms were assessed between 33 and 37 weeks of gestation, women with low depressive symptoms reported significantly lower PBQ scores at 4–6 weeks postpartum compared with those with high depressive symptoms. Depressive symptoms measured at 4–6 weeks postpartum showed the most stable pattern of associations. At this time point, women with low depressive symptoms exhibited lower PBQ scores than those with high symptom severity, while women with moderate symptoms also reported significantly fewer bonding difficulties than those with high depressive symptoms. These group differences persisted at both 6 and 12 months postpartum. A similar pattern emerged when depressive symptoms were assessed at 6 months postpartum. Lower PBQ scores at 4–6 weeks postpartum were observed among women with low depressive symptoms compared with those with high symptoms. At 6 and 12 months postpartum, women with low depressive symptoms reported significantly fewer bonding difficulties than both the high- and moderate-depression groups. Finally, depressive symptoms assessed at 12 months postpartum were also associated with concurrent and earlier bonding outcomes. At both 6 and 12 months postpartum, women with low depressive symptoms demonstrated significantly lower PBQ scores compared with those reporting high depressive symptom severity, while differences between the low- and moderate-depression groups were additionally observed at 12 months postpartum.

Multiple regression analyses were performed to identify predictors of bonding difficulties (PBQ) at three time points (T3, T4, and T5). The regression assumptions were satisfied, with residual analysis confirming linearity, homoscedasticity, and normality. No data imputation was utilized. In all models, maternal depressive symptoms measured at T3 (EPDS T3) emerged as the most consistent and significant predictor of bonding quality. At T3 (n = 92), the model was statistically significant (F(10, 38) = 3.388, *p* = 0.003) and accounted for 33.2% of the variance (Adjusted R2 = 0.332). The only significant predictor was the EPDS T3 score (B = 1.187; 95% CI [0.533, 1.841]; *p* < 0.001). At T4 (n = 110), the model remained significant (F(10, 73) = 2.707, *p* = 0.007), explaining 17.1% of the variance (Adjusted R2 = 0.171). Beyond the continued impact of depressive symptoms (B = 0.721; 95% CI [0.335, 1.107]; *p* < 0.001), parity was found to be a significant factor. Specifically, women in their third pregnancy (B = −6.904; 95% CI [−13.636, −0.172]; *p* = 0.045) and fourth pregnancy (B = −11.942; 95% CI [−23.365, −0.519]; *p* = 0.041) reported significantly lower PBQ scores compared to the reference group. At T5 (n = 115), the model was also significant (F(10, 69) = 2.796, *p* = 0.006), with an Adjusted R2 of 0.185. Similar to previous time points, the EPDS T3 score was the significant predictor of bonding (B = 0.707; 95% CI [0.380, 1.035]; *p* < 0.001). Other variables, including age, education, previous psychiatric treatment, perinatal anxiety, and resilience, did not reach statistical significance in any of the analyzed models. Detailed results for each model are presented in the Supplementary Material (Tables S10–S12).

The Generalized Estimating Equation (GEE) analysis revealed a significant temporal improvement in bonding, with Postpartum Bonding Questionnaire (PBQ) scores decreasing significantly at T4 (β = −0.21; 95% CI: −0.35 to −0.07; *p* = 0.003) and T5 (β = −0.28; 95% CI: −0.43 to −0.12; *p* < 0.001) relative to T3 baseline. Multivariable analysis identified several critical predictors of bonding quality. Postnatal depression (EPDS at T3) was strongly associated with higher PBQ scores, indicating poorer bonding across all time points (β = 0.05; 95% CI: 0.03 to 0.07; *p* < 0.001). Conversely, higher levels of maternal resilience (KOP-26) were associated with significantly better bonding outcomes (β = −0.01; 95% CI: −0.01 to −0.002; *p* = 0.012). Furthermore, mothers in their subsequent pregnancies exhibited significantly lower PBQ scores compared to those with first pregnancy (β = −0.28; 95% CI: −0.50 to −0.07; *p* = 0.011). Demographic factors, including age and education level, did not significantly contribute to the model. A parsimonious model was also tested (QIC = 2311.2), and the primary predictors remained stable and significant; however, the full adjusted model (QIC = 2318.4) is presented here for clinical transparency (Tables S13 and S14).

## 4. Discussion

Perinatal bonding difficulties constitute a clinically relevant phenomenon observed across diverse populations. Previous research indicates that impairments in the maternal–infant bond are most common in the early postnatal period, with prevalence estimates ranging from approximately 6% to 41% of mother–infant dyads, depending on assessment timing and measurement criteria [38]. The COVID-19 pandemic introduced additional stressors associated with an increased risk of bonding difficulties; during this period, impaired mother–infant bonding, as measured by PBQ total scores, was reported in 13.4% of mothers, including severe disturbances in 3.6% [39,40]. Evidence regarding bonding in the context of armed conflict remains limited, although exposure to war has been shown to disrupt early bonding processes [41]. To date, studies directly comparing the prevalence of perinatal bonding difficulties in Poland across pre-pandemic, pandemic, and war-related periods are lacking, representing an important gap in the literature.

In the present study, postnatal bonding difficulties were most pronounced in the early postpartum period, particularly at 4–6 weeks after birth, affecting approximately one-quarter of mothers, and declined at 6 and 12 months postpartum. This pattern is consistent with evidence indicating that bonding disturbances are most common shortly after childbirth and tend to attenuate over time [42,43]. In line with this pattern, longitudinal evidence suggests a progressive decline in at-risk bonding across the postpartum period, whereas difficulties persisting beyond mid-infancy may be associated with later disturbances in the mother–child relationship [44,45].

Interestingly, no significant associations were observed between postnatal bonding and sociodemographic or clinical characteristics, including maternal age, education, place of residence, pregnancy complications, psychiatric treatment history, mode of delivery, or infant feeding method. Parity was the only exception, emerging as a significant predictor in the longitudinal GEE model, with mothers in subsequent pregnancies showing lower PBQ scores compared to first-time mothers. This finding contrasts with previous studies that have identified specific demographic or obstetric risk factors for impaired mother–infant bonding [6,7,8,10]. However, the existing literature remains inconsistent, with several studies similarly reporting no associations with age, feeding practices, or delivery mode, and mixed evidence regarding parity and birth experience [8,46,47,48,49]. The impact of sociodemographic and environmental factors on perinatal bonding therefore remains unclear. One possible explanation for the absence of significant associations in the present study is the prominent role of maternal mental health, as elevated depressive symptoms, which are known to be associated with difficulties in the mother–infant relationship and were prevalent in the studied cohort, may have been more salient than sociodemographic or obstetric characteristics.

In this context, postpartum depression emerges as a well-established risk factor for difficulties in the mother–infant bond, particularly during periods of heightened psychosocial stress [31,50]. Evidence from multiple populations, including Polish samples assessed shortly after delivery, indicates that higher levels of maternal depressive symptoms, especially when severe, are consistently associated with greater bonding difficulties [38,51,52]. Accordingly, the association between EPDS scores and postnatal bonding observed in the present study reflects a robust and widely replicated pattern. Depressive symptoms such as low mood, reduced energy, and feelings of guilt or low self-esteem may interfere with sensitive maternal responsiveness and early mother–infant interactions, thereby contributing to impaired bonding [18,19,38].

Our findings indicate that depressive symptoms were most pronounced in the early postpartum period, particularly at 4–6 weeks after birth, and declined by 6 and 12 months postpartum. This pattern aligns with studies reporting an early postnatal peak in depressive symptoms followed by a gradual decrease at the population level, although substantial heterogeneity in symptom trajectories has been documented, including stable low, increasing, and transient patterns [53,54,55]. The evidence further suggests that the association between maternal depressive symptoms and mother–infant bonding may emerge very early after childbirth and that symptom improvement is accompanied by better bonding outcomes [38,52,56,57]. Nevertheless, reports of elevated depressive symptoms and bonding difficulties extending into the second year postpartum indicate that vulnerability may persist beyond early infancy, underscoring the importance of sustained monitoring and continuity of perinatal mental health support [51].

In the present study, a weak positive correlation was observed between PBQ scores and labor anxiety (LAQ) at 4–6 weeks postpartum. Consistent with previous research, including studies conducted in Polish samples, higher levels of childbirth-related anxiety are associated with an increased risk of postnatal bonding difficulties, such as negative feelings toward the infant and reduced emotional involvement in early caregiving [38,58,59,60]. Moreover, evidence suggests that the impact of labor anxiety on the mother–infant relationship may extend beyond the early postpartum period [61].

A key finding of the present study is that maternal resilience was associated with bonding quality in the longitudinal GEE model, emerging as an independent predictor of PBQ scores across postpartum time points. In unadjusted and descriptive analyses, higher resilience levels were related to fewer bonding difficulties across multiple time points. However, in cross-sectional multivariable models at individual postpartum assessments, the association between resilience and bonding was attenuated once depressive symptoms were taken into account. This pattern suggests that while resilience contributes to bonding quality at the longitudinal population-averaged level, part of its cross-sectional effect may overlap conceptually and statistically with maternal depressive symptoms. In descriptive group comparisons, mothers classified within the high-resilience and high-competence groups consistently reported significantly lower PBQ scores compared with those in the low- and medium-resilience groups across multiple postpartum time points, indicating a consistent association in unadjusted analyses. These findings are in line with prior research demonstrating that greater psychological resilience in the early postpartum period is linked to reduced disturbances in the mother–infant bond [62,63]. In the perinatal context, resilience appears to be underpinned by adaptive cognitive, emotional, and interpersonal resources, including tolerance of uncertainty, positive cognitive appraisal, self-protective behaviors, effective family functioning, and access to supportive psychosocial resources, which may together facilitate sensitive maternal responsiveness and the development of a secure early relational bond [24].

In the present study, no significant associations were found between PBQ scores and anxiety related to war, global events, or the COVID-19 pandemic as assessed by the study-specific measures. Existing evidence on the impact of the COVID-19 pandemic on mother–infant bonding remains mixed, with some studies reporting no detrimental effects of postpartum anxiety, while others suggest impaired bonding in the context of heightened fear and uncertainty [63,64,65,66]. Research conducted in war-affected settings indicates that maternal anxiety and trauma related to armed conflict may disrupt early mother–infant bonding, although direct evidence is limited and findings are not uniform [41,67,68]. Importantly, as the present study was conducted in a country neighboring a conflict zone rather than under conditions of direct warfare, findings from conflict-exposed populations cannot be directly extrapolated, highlighting a gap in the current literature.

## 5. Strengths and Limitations

This study has several limitations. The sample was not fully representative, as most of the 150 participants were highly educated and resided in large urban areas, likely due to online recruitment. The high educational level and predominantly urban residence of the sample may have provided access to greater material, informational, and social resources, potentially buffering the psychological impact of external stressors such as the COVID-19 pandemic or the war in Ukraine. Consequently, the transferability of these findings to populations with lower socioeconomic status or living in more vulnerable contexts may be limited. The study also relied on self-reported pregnancy status and participation after delivery, which could not be independently verified. The lack of data on participants’ personal connections to Ukraine, such as having family members or close friends affected by the war, represents a further limitation, as such factors may have been associated with war-related anxiety. Moreover, citizenship information was unavailable for 23 of the 114 participants in the second study phase, while the remaining participants reported Polish nationality. Another limitation is the small number of participants who completed the COVID-19 anxiety measure during the first study phase (n = 36), reducing the generalizability of findings related to this stressor. In addition, partner support—an important factor associated with mother–infant bonding—was not assessed. The CRAQ, GSAQ, and WAQ were newly developed for this project and have not yet undergone full psychometric validation. Despite these limitations, the longitudinal design represents a major strength. Repeated assessments across five time points enabled the examination of temporal patterns in maternal mental health and bonding during the first postpartum year, providing a more robust and informative perspective than cross-sectional approaches.

## 6. Conclusions

Postnatal bonding is influenced by multiple interacting factors, with bonding difficulties reflecting the interplay of psychological and contextual influences. The present findings indicate that bonding difficulties are most pronounced in the early weeks after birth and gradually decrease over time. Maternal depressive symptoms emerged as the most consistent factor associated with poorer bonding. Maternal resilience, particularly in the domains of family relations and social competence, played a protective role in bonding quality across the postpartum period; however, its influence appeared to be reduced when depressive symptoms were taken into account. Labor-related anxiety was linked to bonding difficulties only in the early postpartum period, suggesting a transient influence. In contrast, anxiety related to the COVID-19 pandemic, the war in Ukraine, and broader socioeconomic conditions showed no direct associations with bonding quality. These findings support the inclusion of routine screening for maternal depressive symptoms during postnatal checkups, particularly in the 4–6-week postpartum period, when depressive symptoms are most strongly associated with bonding difficulties. Overall, these findings underscore the central role of maternal depressiveness in shaping early mother–infant bonding and highlight the importance of early identification for intervention, while pointing to resilience as an important psychological resource that may support bonding.

## Figures and Tables

**Table 1:** Description of time points and measurement tools used in the study.

	T1	T2	T3	T4	T5
Time Point	Before the 33rd Week of Gestation	Between the 33rd and 37th Week of Gestation	Between the 4th and 6th Week Postpartum	At the 6th Month of the Child’s Life	At the 12th Month of the Child’s Life
GDQ	X			X	
EPDS	X	X	X	X	X
LAQ	X	X			
KOP26	X				
CRAQ	X ^a^				
GSAQ	X ^b^				
WAQ	X ^b^				
PBQ			X ^b^	X	X

Notes: ^a^—questionnaire administered only in the first stage of the study, ^b^—questionnaire admin-istered only in the second stage of the study. Abbreviations: GDQ—General Data Questionnaire; EPDS—Edinburgh Postnatal Depression Scale; LAQ—Labor Anxiety Questionnaire; CRAQ—COVID-19 Pandemic-Related Anxiety Questionnaire; GSAQ—Global Situation Anxiety Questionnaire; WAQ—war anxiety questionnaire; PBQ—Postpartum Bonding Scale; KOP26—Resilience Measure Questionnaire.

**Table 2:** Descriptive statistics of the Postpartum Bonding Questionnaire (PBQ) total and subscale scores across postpartum time points.

Total PBQ Score			
	T3 (n = 92)	T4 (n = 110)	T5 (n = 115)
Mean score ± SD	19.7 ± 15.9	15.0 ± 11.1	15.5 ± 9.3
Min–max	1–74	0–59	1–50
0–25	68 (73.9%)	94 (85.5%)	97 (84.3%)
26–39	13 (14.1%)	13 (11.8%)	16 (13.9%)
40 or more	11 (12.0%)	3 (2.7%)	2 (1.7%)
Mother–infant relationship disorders subscale			
	T3 (n = 92)	T4 (n = 110)	T5 (n = 115)
Mean score ± SD	10.3 ± 8.6	9.0 ± 6.2	7.9 ± 5.0
Min–max	0–42	0–31	0–26
0–11	61 (66.3%)	86 (78.2%)	92 (80.0%)
12 or more	31 (33.7%)	24 (21.8%)	23 (20.0%)
Rejection and pathological anger subscale			
	T3 (n = 92)	T4 (n = 110)	T5 (n = 115)
Mean score ± SD	4.6 ± 5.1	3.6 ± 3.7	4.0 ± 3.0
Min–max	0–22	0–18	0–15
0–16	90 (97.8%)	108 (98.2%)	115 (100.0%)
17 or more	2 (2.2%)	2 (1.8%)	0 (0.0%)
Infant-focused anxiety subscale			
	T3 (n = 92)	T4 (n = 110)	T5 (n = 115)
Mean score ± SD	4.6 ± 3.2	3.3 ± 2.4	3.4 ± 2.4
Min–max	0–15	0–10	0–11
0–9	83 (90.2%)	108 (98.2%)	112 (97.4%)
10 or more	9 (9.8%)	2 (1.8%)	3 (2.6%)
Incipient abuse subscale			
	T3 (n = 92)	T4 (n = 110)	T5 (n = 115)
Mean score ± SD	0.2 ± 0.6	0.1 ± 0.3	0.1 ± 0.4
Min–max	0–5	0–2	0–2
0–2	90 (97.8%)	109 (99.1%)	113 (98.3%)
2 or more	2 (2.2%)	1 (0.9%)	2 (1.7%)

**Table 3:** Correlations between postpartum bonding and psychological factors.

	PBQ 4–6 Weeks Postpartum	PBQ 6 Months Postpartum	PBQ 12 Months Postpartum
CRAQ	not measured	ρ = 0.098	ρ = −0.293
		*p* = 0.773	*p* = 0.098
GSAQ	ρ = 0.107	ρ = 0.049	ρ = −0.034
	*p* = 0.309	*p* = 0.627	*p* = 0.760
WAQ	ρ = 0.021	ρ = 0.030	ρ = 0.007
	*p* = 0.846	*p* = 0.767	*p* = 0.950
LAQ < 33 weeks gestation	ρ = 0.253	ρ = 0.136	ρ = 0.149
	*p* = 0.015	*p* = 0.159	*p* = 0.123
LAQ 33–37 weeks gestation	ρ = 0.347	ρ = 0.137	ρ = 0.218
	*p* = 0.014	*p* = 0.331	*p* = 0.132
KOP-26	ρ = −0.345	ρ = −0.289	ρ = −0.285
	*p* < 0.001	*p* = 0.002	*p* = 0.002
KOP-26 family relations subscale	ρ = −0.392	ρ = −0.309	ρ = −0.244
	*p* < 0.001	*p* = 0.001	*p* = 0.009
KOP-26 personal competence subscale	ρ = −0.199	ρ = −0.189	ρ = -0.210
	*p* = 0.058	*p* = 0.048	*p* = 0.024
KOP-26 social competence subscale	ρ = −0.234	ρ = −0.234	ρ = −0.245
	*p* = 0.025	*p* = 0.014	*p* = 0.008
EPDS < 33 weeks gestation	ρ = 0.232	ρ = 0.172	ρ = 0.060
	*p* = 0.026	*p* = 0.074	*p* = 0.538
EPDS 33–37 weeks gestation	ρ = 0.379	ρ = 0.237	ρ = 0.158
	*p* = 0.002	*p* = 0.049	*p* = 0.205
EPDS 4–6 weeks postpartum	ρ = 0.590	ρ = 0.391	ρ = 0.419
	*p* < 0.001	*p* < 0.001	*p* < 0.001
EPDS 6 months postpartum	ρ = 0.450	ρ = 0.494	ρ = 0.473
	*p* < 0.001	*p* < 0.001	*p* < 0.001
EPDS 12 months postpartum	ρ = 0.337	ρ = 0.415	ρ = 0.472
	*p* = 0.004	*p* < 0.001	*p* < 0.001

Abbreviations: PBQ—Postpartum Bonding Questionnaire, CRAQ—COVID-19 Pandemic-Related Anxiety Questionnaire, GSAQ—Global Situation Anxiety Questionnaire, WAQ—War Anxiety Questionnaire, LAQ—Labor Anxiety Questionnaire, KOP-26—Resilience Measure Questionnaire, EPDS—Edinburgh Postnatal Depression Scale. Group comparisons indicated no significant differences in PBQ scores across sociodemographic or clinical characteristics, including age, education level, place of residence, parity, pregnancy complications, psychiatric treatment history, mode of delivery, or infant feeding method.

**Table 4:** Group comparisons of Postpartum Bonding Questionnaire (PBQ) scores across levels of resilience (KOP-26) at postpartum time points.

		PBQ 4–6 Weeks Postpartum		PBQ 6 Months Postpartum		PBQ 12 Months Postpartum	
KOP-26 Total score	Kruskal–Wallis test	H(2) = 15.19, *p* < 0.001		H(2) = 17.66, *p* < 0.001		H(2) = 8.15, *p* = 0.017	
		Mdn [IQR]	† *p* < 0.001 ‡ *p* = 0.015 ‡‡ *p* = 1.000	Mdn [IQR]	† *p* < 0.001 ‡ *p* = 0.003 ‡‡ *p* = 1.000	Mdn [IQR]	† *p* = 0.046 ‡ *p* = 1.000 ‡‡ *p* = 0.106
	Low	19 [10–35]		16 [9–21]		16 [10–25.5]	
	Medium	15 [10–24]		14.5 [7.5–25]		13 [8–16]	
	High	7 [3–11]		5 [3–10.5]		12 [8–14]	
KOP-26 Family relation subscale	Kruskal–Wallis test	H(2) = 10.62, *p* = 0.005		H(2) = 8.71, *p* = 0.013		H(2) = 6.78, *p* = 0.034	
	Low	19.5 [11–33]	† *p* = 0.004 ‡ *p* = 0.029 ‡‡ *p* = 1.000	16.5 [8–24]	† *p* = 0.015 ‡ *p* = 0.032 ‡‡ *p* = 1.000	13.5 [9–22]	† *p* = 0.083 ‡ *p* = 0.038 ‡‡ *p* = 1.000
	Medium	16 [8–31]		15 [7–21]		14.5 [11–23]	
	High	9 [3–13]		6 [3–15]		12 [7–14]	
KOP-26 Personal competence subscale	Kruskal–Wallis test	H(2) = 3.27, *p* = 0.195		H(2) = 9.67, *p* = 0.008		H(2) = 5.48, *p* = 0.064	
	Low	15 [7–35]	† *p* = 0.289 ‡ *p* = 0.294 ‡‡ *p* = 1.000	15 [7–21]	† *p* = 0.039 ‡ *p* = 0.009 ‡‡ *p* = 1.000	15 [9–23]	† *p* = 0.058 ‡ *p* = 0.339 ‡‡ *p* = 0.966
	Medium	16.5 [9.5–24.5]		15 [10–24]		13 [9–22]	
	High	11 [4–21]		7 [3–16]		12 [8–14]	
KOP-26 Social competence subscale	Kruskal–Wallis test	H(2) = 5.95, *p* = 0.051		H(2) = 9.66, *p* = 0.008		H(2) = 10.89, *p* = 0.004	
	Low	19 [1–32]	† *p* = 0.147 ‡ *p* = 1.000 ‡‡ *p* = 0.175	15 [9–21]	† *p* = 0.006 ‡ *p* = 0.069 ‡‡ *p* = 1.000	16 [12–23]	† *p* = 0.028 ‡ *p* = 1.000 ‡‡ *p* = 0.023
	Medium	11.5 [6–24]		13 [7–25]		11.5 [7–17]	
	High	11 [4–23]		5.5 [3–13.5]		11.5 [8–14]	

Notes: Kruskal–Wallis test with post hoc pairwise comparisons using Dunn’s test with Bonferroni correction. H—Kruskal–Wallis test statistic. Statistical significance was set at *p* < 0.05. Abbreviations: PBQ—Postpartum Bonding Questionnaire; KOP-26—Resilience Measure Questionnaire; †—high vs. low; ‡—high vs. medium; ‡‡—medium vs. low.

**Table 5:** Group comparisons of Postpartum Bonding Questionnaire (PBQ) scores across levels of depressive symptoms (EPDS) at postpartum time points.

		PBQ 4–6 Weeks Postpartum		PBQ 6 Months Postpartum		PBQ 12 Months Postpartum	
EPDS < 33 weeks gestation	Kruskal–Wallis test	H(2) = 2.68, *p* = 0.261		H(2) = 1.96, *p* = 0.375		H(2) = 4.97, *p* = 0.083	
		Mdn [IQR]	† *p* = 0.306 ‡ *p* = 1.000 ‡‡ *p* = 1.000	Mdn [IQR]	† *p* = 0.542 ‡ *p* = 0.889 ‡‡ *p* = 1.000	Mdn [IQR]	† *p* = 1.000 ‡ *p* = 0.098 ‡‡ *p* = 0.310
	Low	13 [8–21]		13 [6–18]		15 [11–18]	
	Medium	12 [8–25]		13 [6–20]		11 [8–15]	
	High	18.5 [8–37]		15 [8–24]		15 [11–28]	
EPDS 33–37 weeks gestation	Kruskal–Wallis test	H(2) = 6.67, *p* = 0.036		H(2) = 2.03, *p* = 0.362		H(2) = 2.73, *p* = 0.256	
	Low	12 [8–20]	† *p* = 0.030 ‡ *p* = 0.614 ‡‡ *p* = 1.000	11.5 [5.5–19]	† *p* = 0.974 ‡ *p* = 1.000 ‡‡ *p* = 0.602	13 [9–16]	† *p* = 1.000 ‡ *p* = 1.000 ‡‡ *p* = 0.308
	Medium	13 [7–35]		15.5 [8.5–25]		15 [12–28]	
	High	34 [11–46]		14 [6–24]		14.5 [9.5–23]	
EPDS 4–6 weeks postpartum	Kruskal–Wallis test	H(2) = 33.36, *p* < 0.001		H(2) = 17.41, *p* < 0.001		H(2) = 19.52, *p* < 0.001	
	Low	10 [6–16]	† *p* < 0.001 ‡ *p* = 0.008 ‡‡ *p* = 0.305	11 [6–17]	† *p* < 0.001 ‡ *p* = 0.007 ‡‡ *p* = 1.000	12.5 [8–14]	† *p* < 0.001 ‡ *p* = 0.007 ‡‡ *p* = 1.000
	Medium	13 [9–22]		8 [6–21]		13 [9–17]	
	High	35 [20–47]		24 [16.5–31]		23 [13–28]	
EPDS 6 months postpartum	Kruskal–Wallis test	H(2) = 13.85, *p* = 0.001		H(2) = 22.33, *p* < 0.001		H(2) = 15.37, *p* < 0.001	
	Low	11 [7–20.5]	† *p* = 0.004 ‡ *p* = 0.699 ‡‡ *p* = 0.029	9 [5–16]	† *p* < 0.001 ‡ *p* = 0.634 ‡‡ *p* = 0.002	12.5 [8–15]	† *p* = 0.001 ‡ *p* = 0.391 ‡‡ *p* = 0.038
	Medium	20 [12.5–35]		16 [12–24]		17 [12–25.5]	
	High	28 [19–39]		21 [19–30]		28 [17–29]	
EPDS 12 months postpartum	Kruskal–Wallis test	H(2) = 2.60, *p* = 0.273		H(2) = 8.62, *p* = 0.013		H(2) = 22.68, *p* < 0.001	
	Low	12 [8–24]	† *p* = 0.528 ‡ *p* = 1.000 ‡‡ *p* = 0.766	11 [6–21]	† *p* = 0.024 ‡ *p* = 0.854 ‡‡ *p* = 0.253	13 [8–16]	† *p* < 0.001 ‡ *p* = 0.202 ‡‡ *p* = 0.018
	Medium	19 [11–33]		18 [9–25]		17 [11–23]	
	High	21 [19–33]		24 [19–37]		28 [22–31]	

Notes: Kruskal–Wallis test with post hoc pairwise comparisons using Dunn’s test with Bonferroni correction. H—Kruskal–Wallis test statistic. Statistical significance was set at *p* < 0.05. Abbreviations: PBQ—Postpartum Bonding Questionnaire; EPDS—Edinburgh Postnatal Depression Scale; †—high vs. low; ‡—high vs. medium; ‡‡—medium vs. low.

## Data Availability

The data presented in this study are available on request from the corresponding author.

## Supplementary Materials

The following supporting information can be downloaded at https://www.mdpi.com/article/10.3390/jcm15041451/s1, Table S1: Participant flow and attrition across assessment points; Table S2: Baseline sociodemographic and clinical characteristics of completers and non-completers; Table S3: Items of the COVID-19 Pandemic-Related Anxiety Questionnaire (CRAQ); Table S4: Items of the Global Situation Anxiety Questionnaire (GSAQ); Table S5: Items of the War Anxiety Questionnaire (WAQ); Table S6: Cronbach’s alpha values for the study questionnaires; Table S7: Sample characteristics; Table S8: Symptoms of depression and anxiety; Table S9: Resilience levels; Table S10: Linear regression predicting Postpartum Bonding Questionnaire (PBQ) at T3 (n = 92); Table S11: Linear regression predicting Postnatal Bonding Questionnaire (PBQ) at T4 (n = 110); Table S12: Linear regression predicting Postnatal Bonding Questionnaire (PBQ) at T5 (n = 115); Table S13: GEE model results for predictors of Postnatal Bonding Questionnaire (PBQ) scores trajectory over time; Table S14: Reduced GEE model for predictors of Postnatal Bonding Questionnaire (PBQ) scores trajectory over time [33,69,70,71,72,73,74].
