# Supplementary material for: Postnatal Bonding in the First Year After Birth: The Role of Maternal Depression, Resilience, and Anxiety During the COVID-19 Pandemic and War-Related Stress—A Prospective Cohort Study
Source: J Clin Med. 2026 Feb 12;15(4):1451. doi: 10.3390/jcm15041451 (PMC12942039; doi:10.3390/jcm15041451)
Supplement: Supplementary file 1 [file jcm-15-01451-s001.zip › jcm-4101451-supplementary.pdf]

Journal of Clinical Medicine

Supplementary Materials

Table S1. Participant flow and attrition across assessment points

Study phase	Recruited	<33 weeks gestation	33-37 weeks gestation	4-6 weeks postpartum	6 months postpartum	12 months postpartum
First study phase – available	122	70	51	21	11	33
First study phase – included in analyses	36	29	11	11	11	33
Second study phase – available	152	137	97	107	99	82
Second study phase – included in analyses	114	114	76	92	99	82

Table S2. Baseline sociodemographic and clinical characteristics of completers and non-completers

Variable	Completers (n=71)	Non-completers (n=43)	Comparison		
	Frequency (%)	Frequency (%)	*x2*	*p*	
**Education**	71	43	0.024	0.876	
Secondary	6 (8.5%)	4 (9.3%)			
High	65 (91.5%)	39 (90.7%)			
**Place of residency**	71	43	0.972	0.615	
City with over 100 000 residents	45 (63.4%)	29 (67.4%)			
City up to 100 000 residents	11 (15.5%)	8 (18.6%)			
Rural area	15 (21.1%)	6 (13.9%)			
**Previous psychiatric treatment**	71	43	**7.480**	**0.006**	
Yes	29 (40.8%)	7 16.3%)			
No	42 (59.2%)	36 (83.7%)			
**Previous pregnancies**	71	43	3.662	0.300	
0	36 (50.7%)	14 (32.6%)			
1	18 (25.4%)	14 (32.6%)			
2	11 (15.5%)	10 (23.3%)			
3	6 (8.5%)	5 (11.6%)			
**Number of children**	71	43	0.777	0.678	
0	39 (54.9%)	20 (46.5%)			
1	26 (36.6%)	19 (44.2%)			
2 or more	6 (8.5%)	4 (9.3%)			
**Pregnancy complications**	71	43	1.660	0.198	
Yes	17 (23.9%)	6 (14.0%)			
No	54 (76.1%)	37 (86.0%)			
**Mode of delivery**	71	21	5.849	0.119	
Vaginal delivery	21 (29.6%)	2 (9.5%)			
Assisted vaginal delivery	15 (21.1%)	6 (28.6%)			
Elective cesarean section	16 (22.5%)	9 (42.9%)			
Emergency cesarean section	19 (26.8%)	4 (19.0%)			
	**Mean (SD; range)**	**Mean (SD; range)**	**U**	**Z**	***p***
**Age**	71<br>32.1 (4.2;22-43)	43<br>31.9 (4.3;23-41)	1468.5	0.336	0.737
**GSAQ**	71<br>16.9 (8.0;2-38)	43<br>15.1 (8.4;2-35)	1307.0	1.280	0.200
**WAQ**	71<br>32.0 (9.4;11-60)	43<br>32.5 (11.2;14-62)	1521.5	0.026	0.979
**LAQ T1**	71<br>16.5 (5.6;1-26)	43<br>14.9 (5.5;2-24)	1287.0	1.397	0.161
**LAQ T2**	40<br>14.6 (6.7;0-26)	18<br>14.4 (7.4;0-27)	355.5	0.067	0.946
**KOP-26**	71	43	1372.5	-0.897	0.369

	94.1 (16.4;41-128)	96.1 (19.7;31-127)			
**EPDS T1**	71<br>10.5 (6.5;0-26)	43<br>9.5 (6.0;0-25)	1385.0	0.824	0.401
**EPDS T2**	53<br>8.6 (5.8;0-20)	23<br>9.2 (6.6;0-22)	585.5	-0.266	0.790
**EPDS T3**	71<br>9.5 (5.9;0-22)	21<br>8.8 7.3;0-26)	674.0	0.660	0.508
**EPDS T4**	62<br>7.6 (5.9;0-29)	37<br>6.8 (4.8;0-16)	1091.0	0.401	0.687
**EPDS T5**	71<br>7.6 (5.9;0-24)	11<br>59 (3.2;2-12)	334.0	0.762	0.445
**PBQ T3**	71<br>21.2 (16.8;1-74)	21<br>14.6 (11.0;2-37)	571.5	1.614	0.106
**PBQ T4**	62<br>17.4 (12.4;0-59)	37<br>11.4 (6.9;0-26)	**825.0**	**2.325**	**0.020**

Abbreviations: GSAQ – Global situation Anxiety Questionnaire, WAQ – War Anxiety Questionnaire, LAQ – Labor anxiety Questionnaire, KOP-26 – Resilience Measure Questionnaire, EPDS – Edinburgh Postnatal Depression Scale, PBQ – Postnatal Bonding Questionnaire, SD – standard deviation, χ^2 – chi-square test, U – Mann–Whitney U test, Z – standardized test statistic, n – number of participants; p – significance level. Statistical significance was set at $p < 0.05$ and is indicated in bold.

The General Data Questionnaire collected information on socio-demographic and medical history variables, including age, education level, place of residence, number of previous pregnancies, number of children, history of prior and current psychiatric treatment, and pregnancy complications. Additionally, the postpartum questionnaire collected information in both phases of the study on whether the child was being breastfed, while data on the mode of delivery was recorded only during the second phase.

The Edinburgh Postnatal Depression Scale (EPDS) is a self-report tool for detecting depressive symptoms in postpartum women, also applicable during pregnancy. It consists of 10 items assessing emotional state over the past week, each scored 0–3, with total scores ranging from 0 to 30 [69]. The validated Polish version uses a cut-off of 13/14, with high sensitivity (96%) and specificity (93%) [70]. Based on BC Reproductive Mental Health Program guidelines, scores were categorized as: 0–8, 9–13, and ≥14 [71].

The Labor Anxiety Questionnaire (LAQ) assesses childbirth-related anxiety in pregnant women, focusing on attitudes toward labor and associated fears. It consists of 9 items scored 0–3, with total scores of 0–27. Scores indicate anxiety levels: 0–13 (low), 14–15 (slightly elevated), 16–17 (high), and ≥18 (very high) [72].

The Resilience Measure Questionnaire (org. Kwestionariusz Oceny Prężności, KOP-26) assesses adult resilience across three subscales: Family Competence (KR), reflecting support and strength from family relationships; Personal Competence (KO), indicating self-efficacy and emotional regulation; and Social Competence (KS), measuring interpersonal skills and social support. It includes 26 items rated on a 5-point Likert scale (1 = "strongly disagree" to 5 = "strongly agree"), with total scores ranging from 26 to 130 [73].

The COVID-19 Pandemic-Related Anxiety Questionnaire (CRAQ), developed for the first stage of research, along with the Global Situation Anxiety Questionnaire (GSAQ) and The War Anxiety Questionnaire (WAQ), created for the second stage, are original tools designed by an interdisciplinary team of psychiatrists and psychologists specifically for this study.

The CRAQ consists of 14 items in two parts: the first (4 items) assesses COVID-19-related concerns; the second (10 items) evaluates changes in daily life and related attitudes. The first part uses a 10-point scale (1 = lowest, 10 = highest anxiety), and the second a 6-point Likert scale (1 = "strongly disagree," 6 = "strongly agree"). Total scores range

from 6 to 100. The CRAQ shows good reliability (Cronbach's alpha = 0.79), confirming its usefulness in measuring COVID-19-related anxiety [74].

Table S3. Items of the COVID-19 Pandemic-Related Anxiety Questionnaire (CRAQ)

The COVID-19 Pandemic-Related Anxiety Questionnaire (CRAQ)	
**1.How much are you afraid of the COVID-19 infection?**	10-point scale<br>1 = lowest anxiety<br>10 = highest anxiety
2. How much are you worried that exposure to COVID-19 will harm your unborn/newborn baby?	
3. How much are you afraid of the separation from the loved ones while you are in hospital?	
4. How much are you afraid of the separation from the new-born baby after delivery?	
5. I have limited my contacts with my family and friend due to concerns about mine and my baby's health.	6-point Likert scale<br>1 = "strongly disagree"<br>6 = "strongly agree"
6. I have limited going out from home (e.g. for shopping/walking) due to concerns about my own and my child's health.	
7. I have limited contacts with healthcare professionals due to concerns about getting infected.	
8. I am frustrated when I see people disobeying social distancing rules or people not wearing masks.	
9. When I see someone standing closer than 2 m from me, I move away.	
10. I feel anxious when I see current data about new COVID-19 infections and deaths caused by SARS-CoV-2 virus.	
11. I am afraid that I or my partner will suffer or die because of SARs-CoV-2.	
12. My heart beats faster when I watch news related to COVID-19 pandemic.	
13. I stress out when I hear reports from people who severely suffered from COVID-19.	
14. I cannot sleep because I am worried that something bad will happened to me or my baby due to COVID-19.	

The GSAQ assesses situational anxiety related to societal issues via four items on personal safety amid economic challenges, inflation, the migration crisis, and armed conflict. Responses use a 10-point scale (1 = no concern, 10 = highest anxiety). The tool shows high reliability (Cronbach's alpha = 0.84), supporting its use in analyzing links between societal factors and mental health [33].

Table S4. Items of the Global Situation Anxiety Questionnaire (GSAQ)

The Global Situation Anxiety Questionnaire (GSAQ)	
**1.How concerned are you about your safety in the context of the armed conflict threat?**	10-point scale<br>1 = lowest anxiety<br>10 = highest anxiety
2. How concerned are you about your safety in the context of the migration crisis threat?	
3. How concerned are you about your safety in the context of inflation?	
4. How concerned are you about your safety in the context of economic instability and unemployment?	

The WAQ includes 11 items assessing situational anxiety related to armed conflict, particularly the war in Ukraine. It covers geopolitical impact on personal life, media engagement, conflict-related discussions, contingency planning, and emotional responses. Items are rated on a 6-point Likert scale (1 = "strongly disagree," 6 = "strongly agree"), with total scores ranging from 11 to 66. The WAQ shows good reliability (Cronbach's alpha = 0.81), supporting its use in examining war-related anxiety and mental health [33].

Table S5. Items of the War Anxiety Questionnaire (WAQ)

The War Anxiety Questionnaire (WAQ)	
**1. I follow the developments in Ukraine.**	6-point Likert scale<br>1 = "strongly disagree"<br>6 = "strongly agree"
2. I repeatedly check the current news throughout the day that concerns geopolitical situations.	
3. I am afraid that the war will directly affect me or my family.	
4. I check the news slightly more often than usual.	
5. I cannot sleep if I do not read about what is currently happening in the world.	
6. I am postponing life plans due to the current situation in the world.	
7. I have a detailed action plan in case the armed conflict escalates.	
8. I feel anxious when I see current reports about the number of new victims in Ukraine.	
9. I often talk with my family and close ones about the current situation in the world.	

10. My thoughts are constantly occupied with issues related to the armed crisis in Ukraine.
11. Nothing in my life has changed in connection with the current geopolitical situation.

Table S6. Cronbach's Alpha Values for the Study Questionnaires

Questionnaire (Time Point)	Cronbach's Alpha (α) for our study
EPDS T1	0.89
EPDS T2	0.89
EPDS T3	0.90
EPDS T4	0.88
EPDS T5	0.88
LAQ T1	0.82
LAQ T2	0.90
KOP-26	0.93
CRAQ	0.84
GSAQ	0.80
WAQ	0.82
PBQ T3	0.93
PBQ T4	0.90
PBQ T5	0.87

Table S7. Sample characteristics.

	N=150, Number (%)
**Age (years)**	
Mean ± SD	31.3 ± 4.3
Min-max	20 – 43
**Education**	
High	137 (91.3%)
Secondary	13 (8.7%)
**Place of residency**	
City with over 100 000 residents	95 (63.3%)
City up to 100 000 residents	25 (16.7%)
Rural area	30 (20.0%)
**Previous pregnancies**	
0	67 (44.7%)
1	42 (28.0%)
2	29 (19.3%)
3	8 (5.3%)
4 or more	4 (2.7%)
**Number of children**	
0	76 (50.7%)
1	61 (40.7%)
2 or more	13 (8.7%)
**Complications of the current pregnancy**	
Yes	35 (23.3%)
No	115 (76.7%)
**Previous psychiatric treatment**	
Yes	48 (32.0%)
No	102 (68.0%)
**Mode of delivery**	
Vaginal delivery	23 (15.3%)
Assisted vaginal delivery	21 (14.0%)
Elective cesarean section	25 (16.7%)
Emergency cesarean section	23 (15.3%)
Missing	58 (38.7%)
**Method of infant feeding**	
Exclusive breastfeeding	88 (58.7%)

https://doi.org/10.3390/xxxxx

Combination feeding	62 (41.3%)

Table S8. Symptoms of depression and anxiety

EPDS					
	**T1 (n=143)**	**T2 (n=87)**	**T3 (n=103)**	**T4 (n=110)**	**T5 (n=115)**
**Mean score ± SD**	9.8 ± 6.0	8.8 ± 5.9	9.5 ± 6.2	7.3 ± 5.4	7.3 ± 5.3
**Min-max**	0 – 26	0 – 22	0 – 26	0 – 29	0 – 24
**0-8**	67 (46.9%)	47 (54.0%)	52 (50.0%)	68 (61.8%)	79 (68.7%)
**9-13**	39 (27.3%)	18 (20.7%)	21 (20.2%)	29 (26.4%)	23 (20.9%)
**14 or more**	37 (25.9%)	22 (25.3%)	30 (29.8%)	13 (11.8%)	13 (11.3%)
**LAQ**					
	**T1 (n=143)**	**T2 (n=65)**	**T3**	**T4**	**T5**
**Mean score ± SD**	17.2 ± 5.7	15.4 ± 7.1	not measured	not measured	not measured
**Min-max**	1 – 26	0 – 30			
**0-13**	33 (23.1%)	26 (40.0%)			
**14-15**	18 (12.6%)	7 (10.8%)			
**16-17**	19 (13.3%)	4 (6.2%)			
**18 or more**	73 (51.0%)	28 (43.1%)			

**CRAQ**		**GSAQ**		**WAQ**	
**T1 (n=36)**		**T1 (n=114)**		**T1 (n=114)**	
**Mean score ± SD**	59.5 ± 15.7	**Mean score ± SD**	16.2 ± 8.2	**Mean score ± SD**	32.2 ± 10.0
**Min-max**	20 – 85	**Min-max**	2 – 38	**Min-max**	11 – 62
**0-20**	1 (2.8%)	**0-10**	30 (26.3%)	**0-26**	32 (28.1%)
**21-40**	4 (11.1%)	**11-16**	29 (25.4%)	**27-32**	32 (28.1%)
**41-60**	11 (30.6%)	**17-22**	30 (26.3%)	**33-38**	24 (21.1%)
**61-80**	16 (44.4%)	**23 or more**	25 (21.9%)	**39 or more**	26 (22.8%)
**81 or more**	4 (11.1%)				

Abbreviations: EPDS – Edinburgh Postnatal Depression Scale, LAQ – Labor Anxiety Questionnaire, GSAQ – Global Situation Anxiety Questionnaire, WAQ – War Anxiety Questionnaire, T1 – time point 1, T2 – time point 2, T3 – time point 3, T4 – time point 4, T5 – time point 5.

Table S9. Resilience Levels

**Total score (WO)**					
			**N=150, Number (%)**		
**Mean score ± SD**			95.0 ± 17.6		
**Min-max**			31 – 128		
**0-97**			79 (52.7%)		
**98-109**			44 (29.3%)		
**110 or more**			27 (18.0%)		
**Personal competence (KO)**		**Social competence (KS)**		**Family relation (RR)**	
**Mean score ± SD**	33.3 ± 7.7	**Mean score ± SD**	17.0 ± 5.5	**Mean score ± SD**	44.6 ± 8.8
**Min-max**	9 – 45	**Min-max**	6 – 29	**Min-max**	12 – 55
**0-32**	57 (38.0%)	**0-17**	77 (51.3%)	**0-43**	53 (35.3%)
**33-39**	62 (41.3%)	**18-22**	50 (33.3%)	**44-51**	65 (43.3%)
**40 or more**	31 (20.7%)	**23**	23 (15.3%)	**52 or more**	32 (21.3%)

Table S10. Linear regression predicting Postpartum Bonding Questionnaire (PBQ) at T3 (n = 92)

Model Summary - PBQ T3

**Model**	**R**	**R^2**	**Adjusted R^2**
H_0	0.000	0.000	0.000
H_1	0.687	0.471	0.332

ANOVA

Model		Sum of Squares	df	Mean Square	F	p
H_1	Regression	6405.802	10	640.580	3.388	0.003
	Residual	7185.259	38	189.086		
	Total	13591.061	48			

Coefficients

Model		Unstandardized	Standard Error	Standardized	t	p	95% CI Lower	95% CI Upper
H_0	(Intercept)	19.245	2.404		8.006	< .001	14.412	24.078
H_1	(Intercept)	30.184	21.304		1.417	0.165	-12.943	73.312
	Age	-0.033	0.551	-0.009	-0.061	0.952	-1.148	1.082
	EPDS T3	1.187	0.323	0.507	3.672	< .001	0.533	1.841
	KOP26	-0.193	0.154	-0.179	-1.254	0.218	-0.504	0.118
	LAQ	-0.038	0.797	-0.007	-0.048	0.962	-1.652	1.575
	Education	-11.337	10.478		-1.082	0.286	-32.547	9.874
	Pregnancy							
	Second	-3.621	5.482		-0.660	0.513	-14.719	7.477
	Third	-9.143	5.138		-1.779	0.083	-19.544	1.258
	Fourth	-21.041	11.432		-1.840	0.074	-44.184	2.102
	Fifth	3.892	15.017		0.259	0.797	-26.508	34.292
	Previous psychiatric treatment	4.970	4.719		1.053	0.299	-4.583	14.523

Abbreviations: PBQ – Postpartum Bonding Questionnaire, T3 – 4-6 weeks postpartum, df = degrees of freedom; F = F-statistic; t = t-statistic, p – p-value, CI – confidence interval, EPDS - Edinburgh Postnatal Depression Scale, KOP-26 – Resilience Measure Questionnaire, LAQ – Labor Anxiety Questionnaire.

Table S11. Linear regression predicting Postnatal Bonding Questionnaire (PBQ) at T4 (n=110)

Model Summary - PBQ T4

Model	R	R^2	Adjusted R^2
H_0	0.000	0.000	0.000
H_1	0.520	0.271	0.171

ANOVA

Model		Sum of Squares	df	Mean Square	F	p
H_1	Regression	2909.930	10	290.993	2.707	0.007
	Residual	7846.213	73	107.482		
	Total	10756.143	83			

Coefficients

Model		Unstandardized	Standard Error	Standardized	t	p	95% CI Lower	95% CI Upper
H_0	(Intercept)	15.786	1.242		12.709	< .001	13.315	18.256
H_1	(Intercept)	12.821	13.614		0.942	0.349	-14.312	39.954
	Age	0.326	0.336	0.111	0.973	0.334	-0.343	0.996
	EPDS T3	0.721	0.194	0.400	3.723	< .001	0.335	1.107
	KOP26	-0.122	0.081	-0.171	-1.504	0.137	-0.285	0.040

	LAQ	-0.002	0.234	-9.061×10^{-4}	-0.008	0.994	-0.468	0.464
	Education	1.244	4.486		0.277	0.782	-7.696	10.185
	Pregnancy							
	Second	-2.921	2.961		-0.986	0.327	-8.821	2.980
	Third	-6.904	3.378		-2.044	0.045	-13.636	-0.172
	Fourth	-11.942	5.731		-2.084	0.041	-23.365	-0.519
	Fifth	-1.856	5.819		-0.319	0.751	-13.452	9.741
	Previous psychiatric treatment	-0.225	2.498		-0.090	0.928	-5.203	4.752

Abbreviations: PBQ – Postpartum Bonding Questionnaire, T4 – 6 months postpartum, , df = degrees of freedom; F = F-statistic; t = t-statistic, p – p-value, CI – confidence interval, EPDS - Edinburgh Postnatal Depression Scale, T3 – 4-6 weeks postpartum, KOP-26 – Resilience Measure Questionnaire, LAQ – Labor Anxiety Questionnaire.

Table S12. Linear regression predicting Postnatal Bonding Questionnaire (PBQ) at T5 (n=115)

Model Summary - PBQ T5

Model	R	R^2	Adjusted R^2
H_0	0.000	0.000	0.000
H_1	0.537	0.288	0.185

ANOVA

Model		Sum of Squares	df	Mean Square	F	p
H_1	Regression	1803.318	10	180.332	2.796	0.006
	Residual	4450.632	69	64.502		
	Total	6253.950	79			

Coefficients

Model		Unstandardized	Standard Error	Standardized	t	p	95% CI Lower	95% CI Upper
H_0	(Intercept)	15.525	0.995		15.607	< .001	13.545	17.505
H_1	(Intercept)	12.356	9.941		1.243	0.218	-7.477	32.188
	Age	0.232	0.240	0.113	0.964	0.339	-0.248	0.711
	EPDS T3	0.707	0.164	0.465	4.307	< .001	0.380	1.035
	KOP26	-0.097	0.065	-0.171	-1.511	0.135	-0.226	0.031
	LAQ	-0.017	0.172	-0.011	-0.098	0.922	-0.360	0.327
	Education	0.553	3.615		0.153	0.879	-6.659	7.765
	Pregnancy							
	Second	-3.342	2.265		-1.475	0.145	-7.862	1.177
	Third	-2.648	2.703		-0.980	0.331	-8.040	2.744
	Fourth	-4.764	4.607		-1.034	0.305	-13.955	4.427
	Fifth	-2.182	6.478		-0.337	0.737	-15.104	10.741
	Previous psychiatric treatment	0.037	1.983		0.019	0.985	-3.919	3.993

Abbreviations: PBQ – Postpartum Bonding Questionnaire, T5 – 12 months postpartum, , df = degrees of freedom; F = F-statistic; t = t-statistic, p – p-value, CI – confidence interval, EPDS -

Edinburgh Postnatal Depression Scale, T3 – 4-6 weeks postpartum, KOP-26 – Resilience Measure Questionnaire, LAQ – Labor Anxiety Questionnaire.

Table S13. GEE model results for predictors of Postnatal Bonding Questionnaire (PBQ) scores trajectory over time

Predictor	B	*SE*	*Z*	*p*	95% CI [Lower, Upper]
**Intercept**	3.06	0.57	5.35	**<0.001**	[1.94, 4.18]
**Time (ref: T3)**					
**T4**	-0.21	0.07	-2.93	**0.003**	[-0.35, -0.07]
**T5**	-0.28	0.08	-3.50	**<0.001**	[-0.43, -0.12]
**Age**	0.008	0.013	0.62	0.533	[-0.017, 0.033]
**High education (ref: secondary)**	0.013	0.18	0.07	0.945	[-0.34, 0.37]
**Subsequent pregnancy (ref = first)**	-0.28	0.11	-2.56	**0.011**	[-0.50, -0.07]
**KOP-26**	-0.008	0.003	-2.51	**0.012**	[-0.014, -0.002]
**EPDS T3**	0.052	0.010	5.36	**<0.001**	[0.033, 0.070]

Abbreviations: B – unstandardized regression coefficient, *SE* – robust standard error, *z* – Wald *z*-statistic, *p* – p-value, CI – confidence interval, T3 – 4-6 weeks postpartum, T4 – 6 months postpartum, T5 – 12 months postpartum, EPDS - Edinburgh Postnatal Depression Scale, KOP-26 – Resilience Measure Questionnaire. Significant *p*-values ($p < .05$) are bolded.

Table S14. Reduced GEE model for predictors of Postnatal Bonding Questionnaire (PBQ) scores trajectory over time

Predictor	B	*SE*	*z*	*p*	95% CI [Lower, Upper]
**Time**					
**T4 vs T3**	-0.21	0.07	-2.93	.003	[-0.35, -0.07]
**T5 vs T3**	-0.28	0.08	-3.51	<.001	[-0.43, -0.12]
**Multiparous (ref: primiparous)**	-0.28	0.11	-2.53	.012	[-0.50, -0.06]
**Maternal depression (EPDS T3)**	0.052	0.010	4.95	<.001	[0.031, 0.072]
**Resilience (KOP-26)**	-0.008	0.003	-2.51	.012	[-0.014, -0.002]

Abbreviations: B – unstandardized regression coefficient, *SE* – robust standard error, *z* – Wald *z*-statistic, *p* – p-value, CI – confidence interval, T3 – 4-6 weeks postpartum, T4 – 6 months postpartum, T5 – 12 months postpartum, EPDS - Edinburgh Postnatal Depression Scale, KOP-26 – Resilience Measure Questionnaire.
